# Long-term outcome of early stage prostate cancer treated with brachytherapy analysis after a mean follow-up of 7 years

**DOI:** 10.1186/2193-1801-3-357

**Published:** 2014-07-15

**Authors:** Weigang Yan, Jian Chen, Yi Zhou, Zhien Zhou, Zhipeng Mai, Zhigang Ji, Hanzhong Li, Fuquan Zhang

**Affiliations:** Department of Urology, Peking Union Medical College Hospital, Chinese Academy of Medical Sciences, Beijing, 100730 China; Department of Radiation Oncology, Peking Union Medical College Hospital, Chinese Academy of Medical Sciences, Beijing, China

**Keywords:** Prostate cancer, Brachytherapy, Biochemical no evidence of disease, Overall survival

## Abstract

**Purpose:**

To investigate the long-term efficacy of ^125^I brachytherapy in early-stage prostate cancer and to identify correlating factors.

**Methods:**

This study included 117 cases of early stage prostate cancer. The patients ranged in age from 51 to 84 years, with a mean of 73 years. The features of the study population were as follows: the PSA ranged from 0.4 to 47.6 ng/ml (median, 14.7); the Gleason score ranged from 4 to 9 (mean, 6.4); the clinical stage ranged from T1b to T2c; and the positive biopsy rate ranged from 0.08 to 1.0 (mean, 0.45). The mean D90 was 142 Gy and ranged from 106 Gy to 170 Gy. The numbers of low-risk, intermediate-risk and high-risk prostate cancer cases were 22, 29 and 66, respectively. The biochemical no evidence of disease (bNED) rate and overall survival were recorded. Factors that correlated with the outcomes were evaluated.

**Results:**

With a mean follow up of 84 months, 33 cases had biochemical recurrence, with a bNED rate of 72%. The overall survival rate was 90%, and the cancer-specific survival rate was 97%. The bNED rates in the low-risk, intermediate-risk and high-risk groups were 86%, 79% and 64%, respectively (P = 0.040). The patients with PSA <20 ng/ml, a positive biopsy rate lower than 0.5, and D90 ≥ 140 Gy had lower biochemical recurrence (P = 0.028, 0.006, 0.009, respectively).

**Conclusions:**

The long-term efficacy of ^125^I brachytherapy in early stage prostate cancer was shown. bNED is related to risk stratification, PSA level, positive biopsy rate and D90.

## Introduction

Brachytherapy is a radical treatment that can achieve the same effect as radical prostatectomy and external beam radiotherapy (EBRT) (Nag et al. 1999; Davis et al. 2012; Park et al. 2013). However, studies of the long-term clinical outcome in large cohorts of prostate cancer patients treated with brachytherapy from mainland China are lacking. A total of 564 patients with localized prostate cancer were treated with brachytherapy from December 2003 to August 2013 in Peking Union Medical College Hospital. Among the 138 patients who underwent brachytherapy before December 2007, 117 patients were followed.

## Materials and methods

### Patient characteristics

A total of 117 consecutive patients with localized prostate cancer treated with brachytherapy were enrolled in this retrospective analysis (Table 1). The mean age of the studied cohort was 73 years, ranging from 51 years to 84 years. All of the patients were diagnosed with prostate cancer based on transperineal ultrasound-guided prostate biopsy. The mean PSA level was 14.7 ng/ml, ranging from 0.4 ng/ml to 47.6 ng/ml. The Gleason score ranged from 4 to 9, with a median of 6. The clinical stage ranged from T1b to T2c. The mean prostate volume was 31 ml, ranging from 13 ml to 69 ml. The mean percent positive core (PPC) was 45%, ranging from 8% to 100%. In each patient, pretreatment CT or MRI of the thorax/abdomen/pelvis and a bone scan showed no seminal vesicle or pelvic lymph node involvement and no distant metastasis. Patients were clinically staged based on a medical history and physical and imaging examinations.

**Table 1:** **Patient characteristics**

Parameters	N (%)
Age (years)	
51-70	36 (31)
71-75	40 (34)
76-84	41 (35)
Clinical stage	
T1b	1 (1)
T1c	18 (15)
T2a	27 (23)
T2b	21 (18)
T2c	50 (43)
PSA (ng/ml)	
0-10.0	33 (28)
10.1-20.0	42 (36)
>20.0	42 (36)
Gleason score	
4	3 (3)
5	13 (11)
6	53 (45)
7	31 (26)
8	12 (10)
9	5 (4)
Prostate volume	
≥30 ml	53 (45)
<30 ml	64 (55)
PPC	
<0.5	69 (59)
≥0.5	48 (41)
EBRT	
Yes	6 (5)
No	111 (95)
D 90	
≥140	78 (70)
<140	33 (30)

The risk stratification of the study population complied with the standard provided by NCCN updated in 2012. The low-risk subgroup with a PSA ≤ 10.0 ng/ml, a Gleason score of 2–6 and Stage T1-T2a included 22 patients; 3 patients in this subgroup received neo-adjuvant androgen deprivation therapy (ADT) to shrink prostate before brachytherapy, but the other 19 patients underwent brachytherapy alone. The intermediate-risk subgroup with 10 ng/ml < PSA ≤ 20.0 ng/ml, a Gleason score of 7 and Stage T2b included 29 patients, all of whom underwent 6 months of ADT after brachytherapy. The high-risk group was defined as those with PSA ≥20.0 ng/ml, a Gleason score of 8–10 and Stage T2c. ADT consisted of a gonadotropin-releasing hormone agonist with an antiandrogen. For the intermediate-risk patients, ADT was administered for 6 months, during 3 months of which, it was administered neoadjuvantly. For the high-risk patients, ADT was administered from the 6-month time point to the 3-year time point, during 3 months of which, it was administered neoadjuvantly. Six of the high-risk patients were combined with EBRT (Table 2).

**Table 2:** **Risk groups and treatment method**

Risk group	Patient number	Brachytherapy only	Brachytherapy + ADT	Brachytherapy + EBRT + ADT
Low	22	19	3	0
Intermediate	29	0	29	0
High	66	0	60	6

### Treatment

Three to 7 days before implantation, patients underwent a volumetric study of the prostate performed by transrectal ultrasound (SONOLINE Adara SLC Ultrasound; Siemens, Munich, Germany). Brachytherapy was performed in patients under epidural anesthesia. ^125^I seeds were accurately introduced in preplanned positions by a brachytherapy stepping unit (Computerized Medical System Inc., St. Louis, MO) with a standard 0.5 cm brachytherapy template placed over the perineum. ^125^I implants were generally prescribed to 145 Gy for monotherapy and 110 Gy was used in combination with EBRT. EBRT was delivered with intensity-modulated radiation therapy.

The intraoperative planning time ranged from 20 to 35 minutes (mean 26 min), and the implantation time ranged from 24 to 48 minutes (mean 31 min). Using real-time US guidance, the radioactive seeds were then placed through the needles with a Mick applicator. The implanted seeds were of particle activity ranging from 0.30 mCi to 0.50 mCi each, with a total activity of 15 mCi to 43.2 mCi for each patient, (mean 25.1 mCi). The D90 of monotherapy ranged from 132 Gy to 170 Gy, and that of combination therapy with EBRT ranged from 106 Gy to 113 Gy, with a mean D90 of 142 Gy.

A KUB was scheduled after the procedure to check the distribution of the implanted seeds. The urinary catheter was withdrawn 1 to 3 days after the procedure. Four to 6 weeks after the implantation, dosimetric analysis was performed by computed tomography (CT). The D90 was calculated in each patient.

### Follow-up

The patients were monitored based on serum PSA measurement monthly during the first 3 months after implantation and at 3-month intervals thereafter. If the PSA level was stable, routine follow-up was scheduled every 6 months for 2 years after the implantation. Complications were recorded. The endpoints of this study included biochemical no evidence of disease (bNED) and overall survival. Biochemical failure was determined using the American Society of Radiation Oncology “Phoenix” definition, based on the current nadir plus 2 ng/ml.

Genitourinary toxicities for hematuria, retention, and incontinence and gastrointestinal toxicities were reported using the Radiation Therapy Oncology Group (RTOG)/European Organisation for Research and Treatment of Cancer (EORTC) long-term toxicity scale.

### Statistical analysis

Survival curves were generated using the Kaplan-Meier method. Log-rank analysis was used for comparisons of outcomes in various subgroups. Proportions were compared with the use of chi-square tests. For all tests, a value of p ≤ 0.05 was considered statistically significant. Statistical analysis was performed using SPSS version 19.0 (SPSS Inc., Chicago, IL).

The enrolled patients consented to the study, which was approved by the ethics research committee of the Peking Union Medical College Hospital, Chinese Academy of Medical Science.

## Results

**Figure 1:**
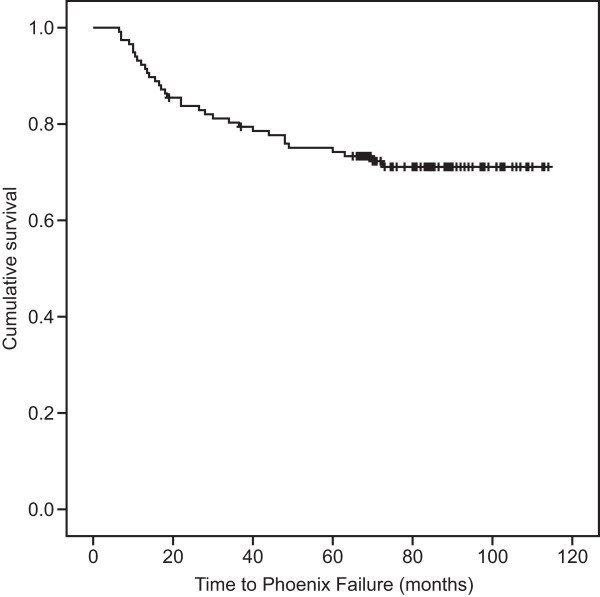
**Biochemical control for all cases, giving a bNED rate of 72%.**

**Figure 2:**
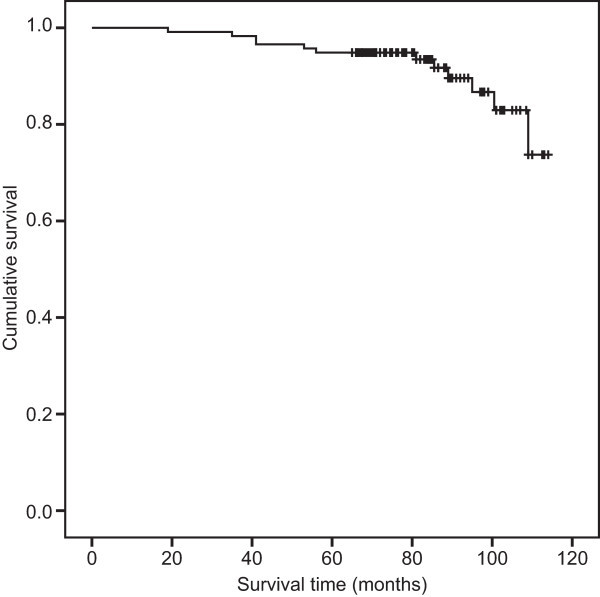
**Overall survival rate for all cases was 90%.**

**Figure 3:**
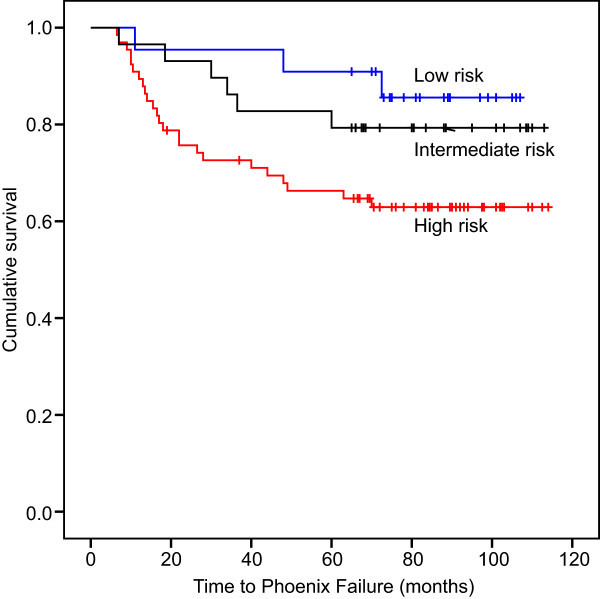
**The bNED rate in low-risk, intermediate - risk and high-risk groups were 86%, 79% and 64% respectively (P = 0.040).**

With a mean follow-up of 84 months and a median follow-up of 86 months (19–114 months), the nadir PSA level ranged from 0 to 1.27 ng/ml (median 0.01 ng/ml). Thirty-three patients had biochemical recurrence, producing a bNED rate of 72% (Figure 1). Twelve patients died, 4 of them from prostate cancer, for an overall survival rate of 90% (Figure 2) and a disease-specific survival rate of 97%.Among the 22 cases of low-risk prostate cancer, 3 experienced biochemical recurrence at 11 months, 48 months and 73 months after brachytherapy; the bNED rate was 86%. No patient died, and the overall survival rate was 100% for low-risk patients. Six of the 29 patients with intermediate-risk disease had biochemical recurrence at 7 months, 19 months, 30 months, 34 months, 37 months and 60 months after the procedure; the bNED rate was 79%. Three deaths occurred because of lung cancer, myocardial infarction and pancreatic cancer at 53 months, 95 months and 109 months after the procedure, producing an overall survival rate of 90% among the intermediate-risk patients. When combined, the low- and intermediate-risk patients had a bNED rate of 82% and an overall survival rate of 94%. Of the 66 high-risk patients, 24 biochemical recurrences were observed between 7 and 70 months after brachytherapy (16 occurred within a year). The bNED rate of this group was 64%. Nine deaths occurred, 4 of which were due to prostate cancer, at 41 months, 41 months, 86 months and 89 months after the procedure. The other 5 cases died from pancreatic cancer, lung cancer, cerebral hemorrhage, pneumonia and renal failure at 19 months, 35 months, 56 months, 81 months and 101 months after the procedure. The overall survival rate of the combined group was 86%. EBRT and ADT were prescribed for patients who biochemically relapsed during the follow-up period. The biochemical control results of the three subgroups were significantly different (P = 0.040) (Figure 3); however, the overall survival rate of the three groups demonstrated no significant difference (P = 0.189).

**Figure 4:**
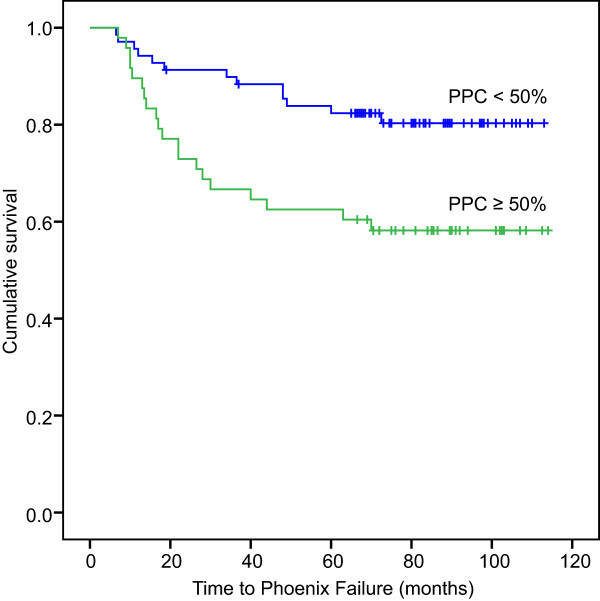
**Patients whose PPC < 50% had less biochemical recurrence likelihood than those whose PPC ≥ 50% (P = 0.006).**

**Figure 5:**
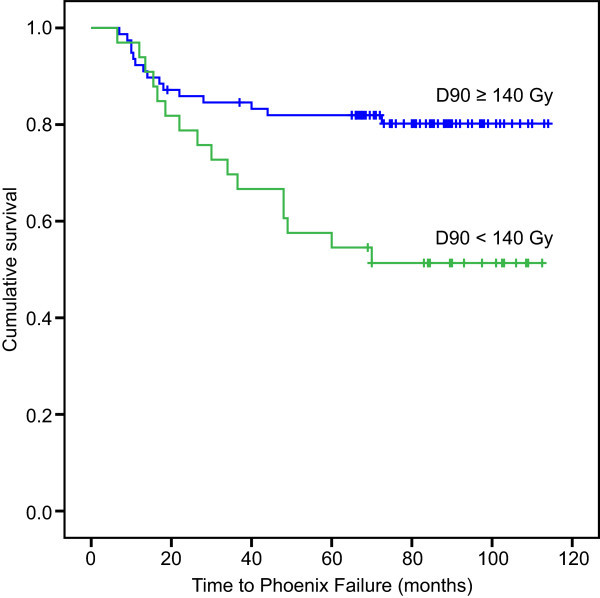
**Patients whose D90 ≥ 140 Gy had better biochemical control than those D90 < 140 Gy (P = 0.009).**

bNED was also examined among the different subgroups (Table 3). Patients whose PPC was lower than 50% had a lower biochemical recurrence likelihood than those whose PPC was higher than 50% (P = 0.006) (Figure 4). Patients who received a D90 higher than 140 Gy had better biochemical control than those who received a D90 lower than 140 Gy (P = 0.009) (Figure 5). Patients with a PSA level lower than 20 ng/ml had a lower biochemical recurrence rate than those with a PSA level higher than 20 ng/ml (P = 0.028). The bNED rate did not significantly differ between clinical stage T1b-T2b and T2c (P = 0.094). Additionally, no significant differences in biochemical relapse were observed in the subgroups with a Gleason score higher or lower than 7 or the subgroups with a prostate volume larger or smaller than 30 ml (P = 0.137, 0.104).

**Table 3:** **bNED rate due to various factors**

Factors	N (bNED rate, %)
PSA	
0-20.0 ng/ml	79 (59/76)
>20.0 ng/ml	60 (25/42)
p-value	0.028
Gleason score	
<7	77 (53/69)
≥7	65 (31/48)
p-value	0.137
Clinical stage	
T1b-T2b	78 (52/67)
T2c	64 (32/50)
p-value	0.094
Prostate volume	
≥30 ml	74 (40/54)
<30 ml	70 (44/63)
p-value	0.740
PPC	
<50%	81 (56/69)
≥50%	58 (28/48)
p-value	0.006
D 90	
≥140 Gy	81 (63/78)
<140 Gy	52 (17/33)
p-value	0.009

RTOG/EORTC graded genitourinary and gastrointestinal toxicities were shown in Table 4. Urinary retention was observed in 11 patients after brachytherapy (9.4%). After urinary catheterization for 1 week, 10 patients were cured. One patient was cured with transurethral prostatectomy because of recurrent episodes of urinary retention. Of the 62% (72/117) potent patients before treatment, 67% (48/72) remained potent. No serious complications such as prostatic rectal fistula were observed.

**Table 4:** **RTOG/EORTC graded genitourinary and gastrointestinal toxicities**

Toxicity	Grage 0-1	Grade 2	Grade 3	Grade 4
Genitourinary				
Retention	106	10	1	0
Hematuria	103	14	0	0
Incontinence	113	4	0	0
Gastrointestinal				
Proctitis	109	6	2	0
Rectal bleeding	108	8	1	0

## Discussion

The radical effect of brachytherapy for localized prostate cancer has been confirmed worldwide. Since the first introduction of ^125^I brachytherapy from western countries to China occurred more than a decade ago, few centers have reported long-term outcomes. To our knowledge, this is one of the largest cohort of prostate cancer patients treated with brachytherapy with the longest follow-up from mainland China reported to date, though the results represent our early experience.

The indication for brachytherapy suggested by the American Brachytherapy Society (ABS) is localized prostate cancer (Nag et al. 1999; Davis et al. 2012). Brachytherapy monotherapy can achieve radical effects for localized low-risk prostate cancer. However, for intermediate- and high-risk patients, EBRT or ADT should be added. Although patients may benefit from the combined therapy in terms of overall survival rate, they also experience increased economic burden, additional complications, and greater inconvenience to their daily lives. For these reasons, the latest ABS guidelines suggest that brachytherapy monotherapy can also be applied in intermediate-risk localized prostate cancer (Davis et al. 2012). Although there is solid evidence indicating that brachytherapy monotherapy achieves the same curative effect as brachytherapy combined with EBRT (Blasko et al. 2000), many clinical centers have added ADT or EBRT routinely for intermediate-risk localized prostate cancer patients (Davis et al. 2012). In our study population, we prescribed 6 months of ADT in intermediate-risk patients. We initially planned to add EBRT for high-risk patients before or after brachytherapy. However, due to the serious urinary track irritation and rectal irritation symptoms associated with the first 6 patients and the relatively optimistic outcomes from other clinics with high-risk patients treated with brachytherapy monotherapy (Merrick et al. 2003; Marshall et al. 2014), we did not perform EBRT in the high-risk patients and opted for brachytherapy monotherapy instead.

Regarding the combination with ADT, we refer to the experience of EBRT combined with ADT: long-term (2–3 years) ADT can improve the biochemical relapse-free survival rate and the overall survival rate of high-risk prostate cancer (Bolla et al. 2009; Hanks et al. 2003). In our study, all of the high-risk patients received ADT, 39 of whom underwent ADT for more than 2 years; ADT in the other 27 cases was administered for less than 2 years for a variety of reasons. Marshall et al.’s study reported that ADT had a benefit in reducing the biochemical recurrence rate after brachytherapy (Marshall et al. 2014). However, Stock has stated that ADT improves FBF only in the setting of lower doses, i.e., a biological effective dose <220 Gy (Stock et al. 2013). The benefit of ADT may primarily be as an enhancer of local control, explaining why high radiation doses can compensate for its absence. Therefore, we prescribed ADT for all high-risk and intermediate-risk patients. Regarding the low-risk patients, the neoadjuvant endocrine therapy was aimed at reducing the volume of the prostate to reduce the interference of the pubic arch.

For early stage prostate cancer, a radical effect can be achieved with brachytherapy, EBRT, brachytherapy combined with EBRT and radical prostatectomy (Davis et al. 2012; Aizer et al. 2009; Kupelian et al. 2004), with similar 5-year biochemical recurrence-free survival rates (Kupelian et al. 2004). Patients can obtain a satisfactory curative effect despite the risk category they fall in (Marshall et al. 2014). Marshall et al.’s analyzed 2495 patients with localized prostate cancer. The 12-year biochemical recurrence-free survival rate was 83% (90% for the low-risk group, 84% for the intermediate-risk group and 64% for the high-risk group). The cancer-specific survival rate was 95%, and the overall survival rate was 70%. A meta-analysis performed by Merrick et al. (2003) showed different clinical outcomes among different centers. The biochemical recurrence-free survival rate for low-, intermediate- and high-risk patients 3 to 7 years after brachytherapy ranged from 85%-96%, 74%-97% and 38%-82%, respectively. With a 7-year follow-up, our study population had an overall biochemical recurrence-free survival rate of 72% (86%, 79% and 64% for the low-, intermediate- and high-risk patients, respectively). The cancer-specific survival rate was 97%, and the overall survival rate was 90%. Compared with previous studies, we obtained similar results in the intermediate-risk patients and relatively poor outcomes in the low-risk patients. We believe the reasons for these findings are as follows. 1. The study enrolled a relatively small study cohort. 2. The study administered a low therapeutic radiation dose. Of the 111 patients in our cohort treated with brachytherapy alone, 33 cases had a D90 < 140 Gy, among which 17 patients had biochemical relapse. The biochemical relapse rate was higher in patients with a D90 < 140 Gy than in those with a D90 ≥ 140 Gy, indicating that the therapeutic radiation dose is related to the cure effect (Stone et al. 2005). 3. In our center, the ^125^I seeds were implanted with a Mick applicator, rather than using a seed strand, thus resulting in a relatively reduction of therapeutic radiation due to seed migration (Lin et al. 2007). However, the seed strand method has not yet been introduced to mainland China.

In general, the factors affecting the effect of brachytherapy can be divided into preoperative, operative and postoperative. The preoperative factors are the PSA level (Potters et al. 2008), Gleason score (Sylvester et al. 2011), risk stratification (Taira et al. 2010), PPC (Taira et al. 2011) and prostate volume (Le et al. 2013). The operative factors are therapeutic radiation dose (Stone et al. 2005) and the proficiency of implantation (Zelefsky et al. 2007), and the postoperative factor is combination with ADT (Marshall et al. 2014) or EBRT (Davis et al. 2012). The results indicated that biochemical recurrence rates differ significantly among groups of patients with different risk factors, showing compliance with results from other centers. However, the overall survival rates among the three groups were of not significantly different, most likely due to our relatively small sample. Based on the analysis of the risk factors, PSA ≥20 ng/ml, PPC ≥50%, and a D90 < 140 Gy were associated with a high biochemical recurrence rate, as suggested by previous studies (Stone et al. 2005; Potters et al. 2008; Taira et al. 2011). Interestingly, we discovered that PPC had a more obvious relationship with the biochemical relapse rate than other factors such as clinical stage or risk stratification. Researchers have also suggested that PPC, being an independent predictive factor, plays a significant role in predicting the biochemical relapse-free survival rate and the overall survival rate in patients undergoing radical prostatectomy (Briganti et al. 2007), EBRT (Spalding et al. 2007) and brachytherapy (Urani et al. 2007).

Urinary retention is the commonly observed complication after brachytherapy for prostate cancer. However, the majority of cases can be improved by urinary catheterization combined with alpha blockers; only a few require TURP intervention. TURP should be delayed 6 months after brachytherapy to avoid urinary incontinence. One of the patients in our study population who had repeated recurrence of urinary retention underwent TURP, with subsequent symptom improvement. Prostate rectal fistula has a relatively low incidence, lower than 1%; however, with a difficult intervention, these cases often require fecal and urinary diversion (Elebrezze & Medich 2003). No prostate rectal fistula was observed in our patients because we were conservative about the radiation dose delivered to the prostate area adjacent to the rectum. The reduced local radiation dose might inevitably have led to relatively poorer treatment effect.

In conclusion, we believe that ^125^I brachytherapy for prostate cancer is an effective, less traumatic method with fewer complications than radical methods of prostate cancer treatment. Further randomized controlled studies with larger samples from multiple centers are needed to verify the efficacy and complications of this method in mainland China.

### Ethical standards

We stated that the study has been approved by the appropriate ethics committee and has therefore been performed in accordance with the ethical standards laid down in the 1964 Declaration of Helsinki and its later amendments. We also stated that all persons gave their informed consent prior to their inclusion in the study.
